# The projected impact of COVID-19 on global surgical care: A case study of International Volunteers in Urology (IVUmed)

**DOI:** 10.7189/jogh.12.03086

**Published:** 2022-12-17

**Authors:** John Lee, Ashley Van Loozen, Danielle Sweeney, Francis Schneck, Christina B Ching

**Affiliations:** 1 Joe R. & Teresa Lozano Long School of Medicine, University of Texas Health Science Center at San Antonio, San Antonio, Texas, USA; 2 International Volunteers In Urology, Austin, Texas USA; 3 Division of Pediatric Urology, University of Pittsburgh, Pittsburgh, Pennsylvania USA; 4 Department of Pediatric Urology, Nationwide Children’s Hospital, Columbus, Ohio USA

## THE DISPARITY IN GLOBAL SURGICAL CARE

Many low- and middle-income countries (LMICs) face immense health care needs with limited available resources, mainly due to the lack of health care providers in specific regions of the world, leading to global inequalities in medical care access. The World Health Organization (WHO) estimates that over 40% of the countries in the world have less than 10 physicians per 10 000 people, and many Sub-Saharan African countries have less than one physician per 10 000 people [1]. In comparison, the United States has 26.1 physicians per 10 000 people, with similar or higher numbers reported in other high-income countries [2].

Unsurprisingly, these health care delivery-related limitations impact surgical care in LMICs, where approximately one third of the world’s population lives, yet where only 6% of surgical procedures are performed [3]. Surgical need is only growing, with an estimated 30% of the global burden of disease being treatable by surgery [4]. Unfortunately, there is a lack of ability to support these needs, with an overall estimate of 5 billion people without adequate and safe access to surgical care. This is worse in LMICs, where up to 90% of individuals have unmet surgical needs [3]. Inequality in access to surgical care has led to high surgically-preventable mortality and morbidity rates in these lower-resourced countries, with not only obvious implications on population health, but economic repercussions as well. The estimated loss of economic productivity between 2015 and 2030 in LMICs due to poorly developed surgical services is US$12.3 trillion [3].

Global humanitarian surgical organizations have attempted to tackle surgical needs in lower-resourced locations, including LMICs. The Current Population Survey estimated that approximately 300 000 people from the United States participated in surgical and non-surgical medical services between 2004 and 2012 [5]. This is likely an underestimation of the true amount of people who have participated, however, as the survey was unable to specifically ascertain volunteer activities and answers typically had pooled responses. With concerns regarding the continuity of care of surgical humanitarian trips and the longevity of their impact [6], there has been a shift to finding sustainable solutions to global health surgical inequalities over the past two decades [7]. Many organizations, including International Volunteers in Urology (IVUmed), have emphasized the importance of in-person training and education of local health care providers, alongside performing surgical procedures on the trips themselves. With the motto of “Teach one, reach many”, IVUmed provides a more permanent commitment to training and supporting local providers, thus providing a strategy for long-term, continuous care to improve surgical access in otherwise under-resourced and under-provided locations.

## IMPACT OF COVID-19 ON SURGICAL CARE – TAXING AN ALREADY CRITICALLY LIMITED RESOURCE

Unfortunately, in-person global humanitarian mission work, including that performed by IVUmed, changed abruptly in March 2020 with the onset of COVID-19 and the resulting international lockdown. COVID-19 affected health care in numerous ways, not only due to the effects of the virus itself, but also due to altered health care patterns. Studies showed that there was a sharp drop in emergency department admissions and increased complications and deaths from emergent diseases like myocardial infarction, aneurysmal subarachnoid haemorrhage, ruptured ectopic pregnancy, diabetic ketoacidosis, and more [8]. This was not an isolated finding restricted to specific countries, but a phenomenon observed worldwide [9]. Additionally, elective surgical cases were pretty universally deferred during this period, resulting in an accumulation of postponed surgical cases. For LMICs, this increasing burden of surgical disease due to COVID-19 stresses an already limited health care system. In some cases, medical and surgical providers succumbed to COVID-19 themselves, removing crucial personnel from the workforce. Compounding all this, at a time when external aid may have been particularly useful, international travel was shut down and global health organizations had to cease all in-person support.

**Figure:**
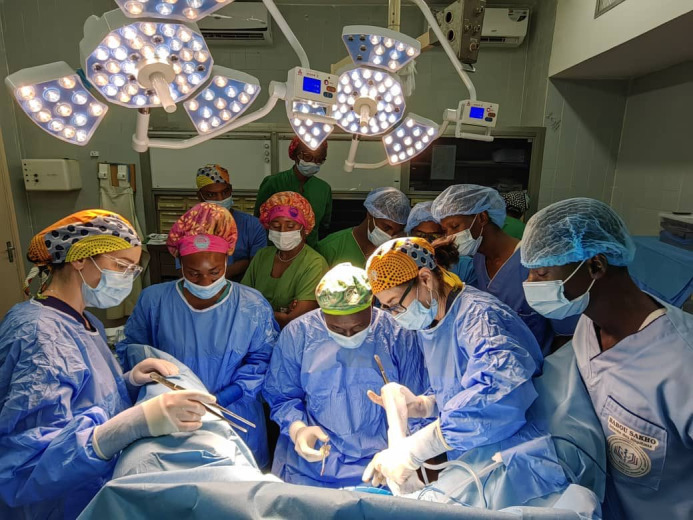
Photo: IVUmed volunteers working together with local partners in Dakar, Senegal under the motto, “Teach one, reach many”. Photo courtesy of Suzette Sutherland, MD; used with permission.

While COVID-19 significantly impacted nearly every aspect of our lives, the toll it has taken on surgical global health organization efforts is nearly unfathomable. Yet, to identify and prepare for the return of travel and resumption of international exchange, it is crucial to identify the loss to global health outreach caused by COVID-19. We at IVUmed aimed to identify the potential loss of productivity due to COVID-19 we experienced as a global health organization in anticipation of the gaps that would exist in surgical care would when global workshops resume.

## CASE STUDY OF IVUMED PRODUCTIVITY LOST DURING COVID-19

To attempt to quantify the loss of halted surgical missions with IVUmed due to the COVID-19 pandemic, we reviewed data related to surgical workshops from the five fiscal years (FY) (April to March) prior to COVID-19. This included metrics on the number of patients seen and surgical cases performed, local surgeons trained, countries visited, and the estimated value of service provided as part of financial impact reporting. The last IVUmed workshop was held from March 5 to 15, 2020, and concludes the FY 2020. No surgical workshops were performed for FY 2021 and through October 31, 2021 (FY 2022). The projected FY loss of productivity for each metric was calculated by averaging the five FYs prior to FY 2021. The total loss since the COVID-19 pandemic was then calculated by the sum of the projected FY 2021 (this value) and that of FY 2022 thus far (7/12^ths^ of this value).

Averaging IVUmed surgical workshops over FY 2016-2020, 23 trips were taken each year to 13 countries. An average of 812 patients were seen annually, with an average of 564 surgical cases performed. On average, 296 local surgeons were involved in each workshop annually. The FY average value of service was US$4 204 217.60, including the cost of surgery and supplies. Projected losses for FY 2021 through October 31, 2021, of FY 2022 are listed in **Table 1**.

**Table 1:** Impact of IVUmed surgical workshops per fiscal year and that lost during the COVID-19 pandemic

Metric	Data per fiscal year prior to COVID-19	Projected loss over fiscal year 2021 and 7/12ths of fiscal year 2022
Number of workshop trips	23	36
Number of local patients seen	812	1286
Number of surgical cases performed	564	893
Number of local surgeons trained	296	469
Average value of service (US$)	4 204 217.60	6 656 667.86

Undoubtedly, COVID-19 has negatively impacted the already critically limited global surgical care provided in LMICs. A simple calculation of lost surgical workshops from a single global health organization attempts to quantify the pandemic’s impact on surgical global health organizations and their under-resourced partners. Although this is an estimate of the impact of COVID-19 on only a single global health program, IVUmed is not unique in that most global health programs were impacted similarly by COVID-19. The total impact of the universal loss of global health services being provided during the COVID-19 pandemic is realistically immeasurable. However, quantifying an estimate of a program’s loss can help prepare for the potential backlog of care and training faced with resuming workshops.

There may be potential strategies to address these deficits, including increasing the frequency of surgical workshops when able to return to in-person exchange. Another strategy may be thoughtful planning to accommodate a larger volume of surgeries performed and an increase in educational opportunities provided in any one trip, possibly through larger than usual volunteer groups. In a similar article assessing the impact of COVID-19 on cleft palate surgeries in LMICs, Wood et al recommended a more proactive approach [10], emphasizing support of local training and infrastructure of LMIC hospitals to provide their own surgical and medical care in a sustainable manner. With less reliance on humanitarian aid missions, there would be less of an impact by possible future global lockdowns.

## REDIRECTING SURGICAL CARE VIRTUALLY

Despite the havoc created by COVID-19 and numerous resulting setbacks, many global surgical organizations (including IVUmed) came up with innovative ways to continue their involvement in global health and providing ongoing training to local partners. IVUmed’s focus during the pandemic lockdown shifted towards virtual education with its local partners. We established a virtual visiting professorship program, where volunteer urologists led live-streamed teaching sessions to partner sites based on requested needs. Local sites simply needed an internet connection to participate. This enabled long-distance interactions amongst lecturers and attendees and the opportunity for live questions. Sites throughout the continent of Africa, East Asia, and the Caribbean participated. These programs were also recorded and catalogued to enable future reference for interested parties. We also implemented a virtual consultation service to enable real-time discussions between volunteer urologic subspecialists with local providers regarding complex patient care. In these ways, we tried to continue to support local teams during a time of immense isolation; and we continue to provide such a program for our partner sites. IVUmed also used time during COVID-19 to build an electronic medical record for surgical workshops to provide better continuity of care.

During the pandemic, we evaluated our processes as an organization, integrated new methods, and found opportunities for improvement. While COVID-19 had devastating effects on patients and providers around the world, it has also demonstrated the resilience of humankind and the ongoing need for global connections and support.
